# Charge Density Waves in Electron-Doped Molybdenum Disulfide

**DOI:** 10.1021/acs.nanolett.1c00677

**Published:** 2021-07-06

**Authors:** Mohammed K. Bin Subhan, Asif Suleman, Gareth Moore, Peter Phu, Moritz Hoesch, Hidekazu Kurebayashi, Christopher A. Howard, Steven R. Schofield

**Affiliations:** † Department of Physics and Astronomy, University College London, WC1E 6BT London, United Kingdom; ‡ Photon Science, Deutsches Elektronen-Synchrotron (DESY), Notkestrasse 85, 22607 Hamburg, Germany; § London Centre for Nanotechnology, University College London, WC1H 0AH London, United Kingdom; ∥ London Centre for Nanotechnology, University College London, WC1H 0AH London, United Kingdom; # Department of Electronic and Electrical Engineering, University College London, WC1E 6BT London, United Kingdom

**Keywords:** Charge density wave, metal−insulator transition, MoS_2_, intercalation, STM, tunneling spectroscopy

## Abstract

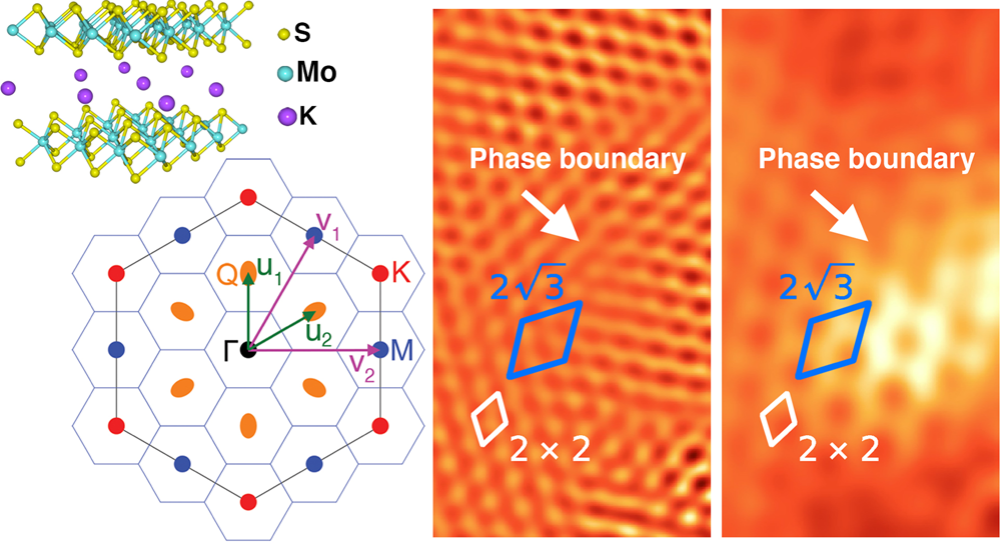

We present the discovery of a charge density wave (CDW) ground state in heavily electron-doped molybdenum disulfide (MoS_2_). This is the first observation of a CDW in any *d*^2^ (column 6) transition metal dichalcogenide (TMD). The band structure of MoS_2_ is distinct from the *d*^0^ and *d*^1^ TMDs in which CDWs have been previously observed, facilitating new insight into CDW formation. We demonstrate a metal–insulator transition at 85 K, a 25 meV gap at the Fermi level, and two distinct CDW modulations, (2√3 × 2√3) R30° and 2 × 2, attributable to Fermi surface nesting (FSN) and electron–phonon coupling (EPC), respectively. This simultaneous exhibition of FSN and EPC CDW modulations is unique among observations of CDW ground states, and we discuss this in the context of band folding. Our observations provide a route toward the resolution of controversies surrounding the origin of CDW modulations in TMDs.

Strongly anisotropic crystals that confine charge carriers to two dimensions exhibit a rich diversity of correlated ground states, including charge density waves (CDWs), spin density waves, and superconductivity. However, despite decades of intense effort, there are still large gaps in our understanding of the mechanisms underpinning the formation and competition between such states. New experimental observations of correlated states can provide litmus tests for competing theoretical models. CDWs are a periodic spatial oscillation of charge density, accompanied by a lattice distortion, that occur in crystalline materials due to electron–electron and electron–phonon interactions.^1−3^ Despite intense investigation, the physics of CDW formation remains a topic of vigorous debate,^4,5^ and the connection to other exotic electronic ground states, most notably superconductivity, remains controversial.^6^

Transition metal dichalcogenides (TMDs) are two-dimensional (2D) layered materials that are tailorable by varying the elemental composition, coordination, symmetry, layer number and separation, and doping.^7,8^ TMDs thus provide excellent opportunities to investigate fundamental condensed matter physics in reduced dimensions. CDWs have been discovered in the semimetallic column 4 (*d*^0^) TMDs, TiSe_2_,^9^ and TiTe_2_,^10^ and in the metallic column 5 (*d*^1^) TMDs VS_2_,^11^ VSe_2_,^12^ NbSe_2_,^4,13^ TaS_2_,^14^ and TaSe_2_.^15^ CDWs have not been previously observed in column 6 (*d*^2^) TMDs, which are typically band semiconductors. However, density functional theory calculations have indicated the possibility of CDW formation in heavily doped bulk^16,17^ and monolayer MoS_2_,^18,19^ and a recent study reported anomalies in the temperature dependence of the sheet resistance in electron-doped MoS_2_, suggesting the possibility of a CDW phase transition.^20^ There are two popular mechanisms for CDW formation in TMDs: Fermi surface nesting (FSN; the favored model in VSe_2_, TaS_2_, and TaSe_2_^3,12^), which requires coupling of the Fermi surface and leads to the opening of a small energy gap centered at the Fermi energy; and momentum-dependent electron–phonon coupling (EPC; favored in NbSe_2_ and VS_2_^11^), which can occur in the absence of strong connections within the Fermi surface. In many cases, not all aspects of the data are adequately described by either model.

MoS_2_ is a band semiconductor, column 6 (*d*^2^) TMD with a trigonal prismatic (2H) ground state (Figure 1c).^7^ The band edges derive from 4*d* orbitals,^7^ and the bulk material has a 1.29 eV indirect band gap^21^ (Figure 1a). In reciprocal space, the valence band maximum is at the zone center (Γ), while the conduction band minimum is located midway along Γ–K, producing a 6-fold degenerate conduction band with electron pockets at the points labeled Q in Figure 1a,b. With electron doping, MoS_2_ undergoes a metal–insulator transition at a free carrier density of *n*_2D_ = 6.7 × 10^12^ cm^–2^ and exhibits a sharp onset of superconductivity at 6.8 × 10^13^ cm^–2^.^22^

**Figure 1:**
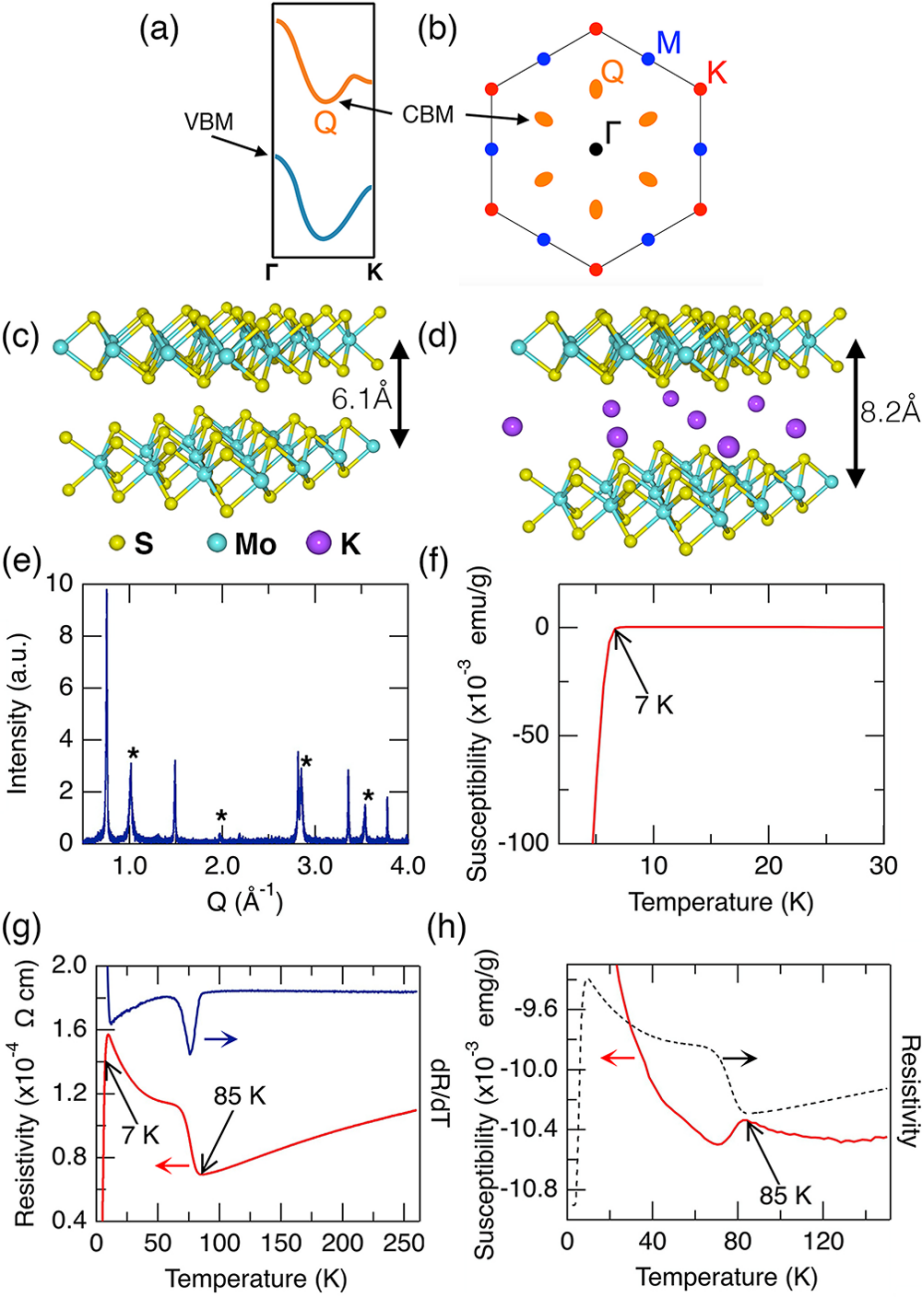
(a) MoS_2_ band structure schematic highlighting valence/conduction band maximum/minimum (VBM/CBM) (adapted from ref (27)). (b) Brillouin zone with high-symmetry points Γ, K, M, and low-symmetry point Q. (c) Crystal structure of MoS_2_ and (d) of K_0.4_MoS_2_. (e) K_0.4_MoS_2_ X-ray diffraction; asterisks denote unintercalated MoS_2_ peaks. (f) Magnetic susceptibility (10 Oersted). (g) Resistivity (red trace) showing metal–insulator (85 K) and superconducting transitions (7 K); d*R*_S_/d*T* is shown on the right axis (blue trace). (h) Magnetic susceptibility (red trace) with a large applied field (10^4^ Oe) with resistivity data overlaid (dashed curve; right axis).

Here, we present the discovery of a CDW ground state in bulk potassium-intercalated (and therefore electron-doped) MoS_2_ with a simultaneous exhibition of FSN and EPC derived modulations. We demonstrate a metal–insulator transition at 85 K, (2√3 × 2√3) R30° and 2 × 2 CDW modulations via atomic-resolution scanning tunneling microscopy (STM) and a 25 meV energy gap at the Fermi level via tunneling spectroscopy. The 2√3 modulation is perfectly matched by a nesting vector connecting the conduction band pockets, while the 2 × 2 modulation matches a theoretically predicted phonon-mode softening at the M point.^18^ We discuss that the two modulations are simple linear combinations of one another, suggesting that the driving mechanisms may be coupled via band folding.

Potassium ions were intercalated into the van der Waals gaps of a bulk MoS_2_ sample using the well-established low-temperature liquid ammonia method.^23^ Briefly, high-quality MoS_2_ crystals (Manchester Nanomaterials) were degassed (523 K, <10^–6^ mbar) then combined with potassium dissolved in liquid ammonia at 218 K. Potassium intercalation (Figure 1d) completed after ∼24 h. X-ray diffraction (XRD) was measured in a reflection geometry (Philips X’Pert) on cleaved samples in an airtight beryllium dome. Magnetic susceptibility measurements (Quantum Design MPMS-7) were performed on samples held in a plastic capsule and sample straw. Four-terminal contacts were attached to the sample using Epotek H21D silver epoxy and transport measurements (Keithley 2400 SMU and Stanford Research Systems SR830) were made on a coldfinger below 10^–5^ mbar with a 1 mA current and 10 Kh^–1^ heating rate. STM measurements (Omicron LT-STM) were performed on samples cleaved under ultrahigh vacuum (<5 × 10^–10^ mbar) at room temperature to produce an atomically clean surface of a bulk intercalated sample, and then cooled to 5.5 K for STM measurement.

XRD confirms the crystalline quality of our samples (Figure 1e); the 00*l* out-of-plane peaks are shifted with respect to their unintercalated positions, demonstrating the expected 2.2 ± 0.1 Å increase in the layer separation.^23^ Magnetic susceptibility and four-terminal resistivity measurements (Figure 1f and g) confirm the onset of superconductivity at *T*_c_ = 7.0 ± 0.5 K.^23,24^ The room temperature resistivity (*R* ∼ 1 × 10^–4^ Ω cm) decreases linearly with temperature (Figure 1g, red trace), as expected.^25^ At 85 K, we find a pronounced step increase of ∼5 × 10^–5^ Ωcm, marking the location of a metal–insulator transition. Also at 85 K we find an abrupt decrease (−0.02 emu/g) in magnetic susceptibility (Figure 1h). These features are characteristic of the opening of an energy gap at the Fermi level, and similar behavior has been attributed to CDW transitions in VS_2_^11^ and TaS_2_.^26^

Figure 2a displays a topographic STM image of a region free from step edges or adatoms, and that is characteristic of images acquired using different samples and tips. The Fourier transform of this image (Figure 2b) exhibits hexagonal spots corresponding to a lattice constant, *a* = 3.14 ± 0.07 Å, in agreement with the calculated lattice constant, 3.176 Å,^28^ confirming that we are imaging the surface sulfur atoms of the cleaved sample. A 0.2 monolayer surface coverage of potassium ions might be expected if half of the potassium ions in the cleaved layer remain on the surface after cleaving. However, we do not observe any surface potassium ions in our images and attribute this to the surface potassium ions diffusing to step edges or elsewhere while the sample is at room temperature before it is loaded into the STM and cooled. Several defects in Figure 2a appear as protrusions several nanometers in diameter superimposed on the surface atomic lattice. Similar defects, attributed to molybdenum vacancies or antisites, have been shown to locally enhance the MoS_2_ interlayer coupling^29,30^

**Figure 2:**
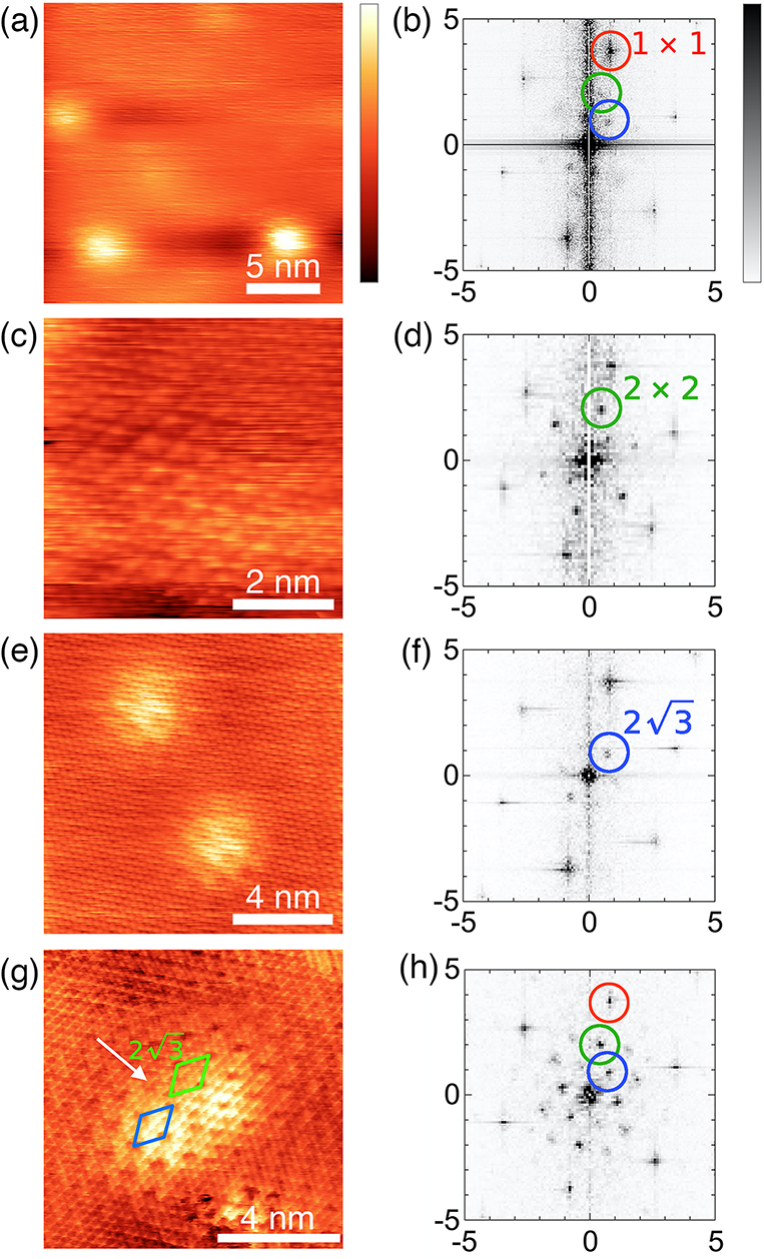
(a,c,e) STM topographic images of K_0.4_MoS_2_ (5.5 K) and (b,d,f) their corresponding 2D Fourier transforms. Bragg spots corresponding to the 1 × 1 surface sulfur lattice are seen in all images, while to a lesser or greater extent 2 × 2 and 2√3 periodicities are also observed (see text). Image parameters: (a) −150 mV, 20 pA, z-range 1.5 nm; (c,e) −100 mV, 30 pA, z-range 700 pm. FFT axes are in nm^–1^ units. (g) Filled-state STM image of a pair of closely spaced defects in K_0.4_MoS_2_. 1 × 1, 2 × 2, and 2√3 periodicities can be seen in the image and its Fourier transform in panel (h). A phase slip boundary (white arrow) exists between the two regions of 2√3 periodicity, which we highlight on panel (g) by adding a blue rhombus to indicate the phase of the 2√3 modulation at the bottom left defect site, and a green rhombus to indicate the 2√3 phase of the top right defect.

Figure 2c shows a higher resolution image acquired with a lower imaging bias magnitude (−100 mV). This image and the corresponding Fourier transform (Figure 2d) reveal a longer-ranged periodicity of 5.8 ± 0.2 Å, i.e., 2 ± 0.2 times the 1 × 1 lattice, which we interpret as a 2 × 2 CDW modulation. We notice that the apparent intensity of the 2 × 2 modulation varies within the image (Figure 2c), suggesting a nearly commensurate CDW phase, as observed in STM images of NbSe_2_^13^ and copper-intercalated TiSe_2_.^31^ Examining again the Fourier transform of the larger area image in Figure 2b, we see the 2 × 2 spots are also faintly visible in this data. Indeed, in all images of the K_0.4_MoS_2_ surface acquired with sufficient resolution we find evidence for a 2 × 2 periodicity to a greater or lesser extent depending on the imaging parameters and location on the surface, suggesting that in K_0.4_MoS_2_ we have a nearly commensurate 2 × 2 CDW state present everywhere throughout the sample surface.

The image in Figure 2e shows an area where two defects are present. Here, we find an additional periodicity, distinct from both the 1 × 1 lattice, and the 2 × 2 modulations. The Fourier transform (Figure 2f) demonstrates that this periodicity is characterized by a 10.4 ± 0.2 Å real space vector, which is rotated 30° with respect to the 1 × 1 lattice. We note that within uncertainties, this periodicity is 2√3 times the 1 × 1 unit cell vector (=10.7 ± 0.4), and we therefore label this as a (2√3 × 2√3)R30° modulation of the K_0.4_MoS_2_ surface. Unlike the 2 × 2 modulation, the 2√3 modulation is strongly enhanced within the vicinity of the defect sites. We found such 2√3 modulations always occur at such defects sites; e.g., the 2√3 spots are visible in Figure 2b due to the defects in the corresponding STM image, and further examples are shown in Figures 2g,h and S1. Modulations of the local density of states in the vicinity of defects can occur due to quasiparticle interference (QPI);^32^ however, we rule out QPI in the present case since the modulation is dispersionless^32^ (see the spatially resolved tunneling spectroscopy presented below). It is possible also to draw comparison to NbSe_2_, where the 3 × 3 CDW modulation was found to persist above *T*_CDW_ in patches surrounding defects.^13^ However, our measurements of defects in K_0.4_MoS_2_ at 5.5, 10, and 77 K show negligible change in the spatial extent of the 2√3 modulation surrounding the defects with temperature (see Supporting Information Figure S1). Moreover, we also observe phase slip boundaries in the 2√3 modulation for closely spaced defects, similar to observations of phase boundaries in CDW modulations seen in NbSe_2_^33^ and TiSe_2_.^34^ We show an example of this in Figure 2g, which shows a filled-state STM image where two defects are present (the corresponding Fourier transform is shown in Figure 2h). The two defects exhibit 2√3 modulations, but these two modulations are not in phase, leading to a phase slip boundary between them, which we highlight with a white arrow in Figure 2g. Further analysis and discussion of this phase slip boundary can be found in Figure S2. The implication of these observations is that the 2√3 modulation can be attributed to a CDW whose enhancement at defect sites is an intrinsic property, and not due to QPI or an incompletely formed CDW phase.

Other non-CDW explanations for the observed 2 × 2 and 2√3 periodicities can be ruled out. The precise arrangement of the potassium ions in K_0.4_MoS_2_ is not known;^23,28^ however, the K_0.4_MoS_2_ stoichiometry^23^ and hexagonal symmetry of the crystal suggests a hexagonal arrangement of the average positions of the potassium ions, with a lattice constant of (5/2)^1/2^*a*. Thus, even allowing for all possible rotational orientations of the potassium ion layer with respect to the top MoS_2_ layer, we can rule out both a direct influence of the potassium ion positions and Moiré interference between the MoS_2_ and potassium ion layers as explanations for either periodicity. Moiré effects due to strain at defect sites can be additionally ruled out by the observation that the characteristic length of the modulation does not vary with distance from the defect center as would be expected for a point-source lattice distortion derived Moiré interference.

Figure 3 presents scanning tunneling spectroscopy (STS) measurements of K_0.4_MoS_2_. Point spectra show a bulk band gap of around 1.2 eV (Figure 3a), consistent with previous measurements^30^ and calculations.^28^ Higher-resolution measurements at the conduction band edge (Figure 3b) show the band minimum is located ∼25 meV below the Fermi level, in agreement with ARPES measurements of potassium-doped MoS_2_,^35^ and a similar band shift in copper intercalated TiSe_2_.^31^

**Figure 3:**
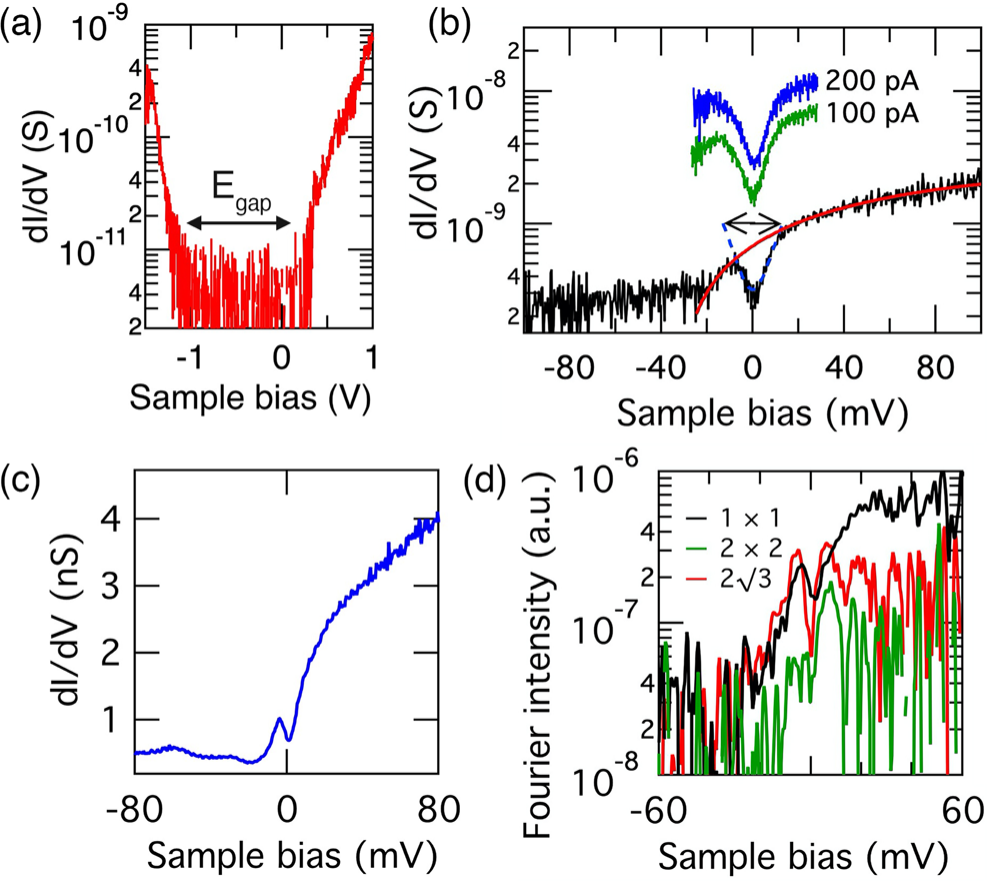
Point tunneling spectroscopy of K_0.4_MoS_2_ highlighting (a) the ∼1.2 eV band gap and (b) the conduction band minimum at −25 meV and gap at the Fermi level; curve fits are to the band edge (red) and energy gap (dashed blue). (c) Spatial average of d*I*/d*V*. (d) Fourier spot intensities for the 1 × 1, 2 × 2, and 2√3 lattice spots (see Figure S3 for corresponding d*I*/d*V* images at ±5 mV). Tunneling spectra were acquired by measuring current as a function of voltage with the feedback loop off. Set-point parameters: (a) −1.5 V, 50 pA; (b) black: −100 meV, 30 pA; green −30 meV, 100 pA; blue: −30 meV, 200 pA. All data 5.5 K, except panel (b) 10 K.

We also find an energy gap at the Fermi level (Figure 3b,c); we measure the gap as the point where a quadratic fit to the gap intersects the conduction band and take the gap to be twice this value, yielding 2Δ = 24 ± 4 meV. There was no measurable difference between spectra taken on or away-from defect sites, demonstrating that the gap occurs across the entire surface.

We have measured spatially resolved conductivity, *g*(*r*, *V*), where *r* is the tip position and *V* is the sample bias (see Figure S3); when integrated over *r* (Figure 3c), this yields good agreement with the point spectroscopy in Figure 3b. We deconvolve contributions associated with the different superlattice modulations by taking a 2D Fourier transform of *g*(*r*, *V*) for each value of bias to obtain conductivity as a function of the reciprocal space vector *q*. We identify in this data the expected 1 × 1, 2 × 2, and 2√3 reciprocal lattice vectors and show the intensity variation of these spots as a function of bias in Figure 3d. The observation that these spots occur at the same values of *q* for each value of bias (see Figure S3) rules out QPI origins for these modulations, and we do not observe any additional spots for any values of the applied bias. The 1 × 1 spot intensity variation (Figure 3d) closely matches the spatially averaged conductance (Figure 3c). In contrast, the 2√3 spots exhibit a gap feature and peaked intensities either side of the Fermi level. The intensity variation of the 2 × 2 spots follow similarly to the 2√3 spots, although the signal-to-noise ratio is insufficient to say conclusively whether or not the gap exists in the 2 × 2 data. Concentrating on the 2√3 curve, which has the better signal-to-noise ratio, we measure peak maxima at ±3.1 ± 0.1 mV, i.e., separated about the Fermi level by a width of 6.2 ± 0.2 mV. Alternatively, if we measure the width from the outer edges of the peaks, we find a width of 26 ± 2 mV. These widths compare well to the gap width estimation from our point spectra in Figure 3b. Thus, the spatially resolved tunneling spectroscopy data shown in Figure 3, and the fact that the Fermi level gap in STS spectra occurs far from defect sites where the 2 × 2 modulation persists but the 2√3 modulation does not, suggest that the energy gap at the Fermi level correlates to both the 2 × 2 and 2√3 spatial modulations observed in our STM and STS images.

Our observations of a metal–insulator transition at 85 K and a 24 ± 4 meV energy gap centered at the Fermi level are suggestive of a FSN-driven CDW in K_0.4_MoS_2_. The necessary FSN vectors exist in the K_0.4_MoS_2_ electronic structure due to the fact that potassium intercalation of MoS_2_ creates electron pockets at the Q points. The length of the vector, **q** (Figure 4a), that connects the Fermi surfaces at Q is |**ΓK**|/2, i.e., , where **b** is the reciprocal lattice vector. There are three pairs of such nesting vectors, each rotated 30° to the 1 × 1 surface sulfur lattice. Thus, the vectors **q** connecting neighboring Q points provide an excellent match to our observed (2√3 × 2√3) R30° modulation in STM and STS data. This provides strong evidence for a FSN-driven 2√3 CDW phase in K_0.4_MoS_2_ that is localized at or enhanced by defects in the MoS_2_ sheets. The reason for this enhancement at defects is that the defects locally enhance the interlayer coupling,^29,30^ resulting in a lowering of the band edge at the Q point and thus an increased occupancy of the bulk-like electron pockets^7^ and a corresponding enhancement of the 2√3 FSN.

**Figure 4:**
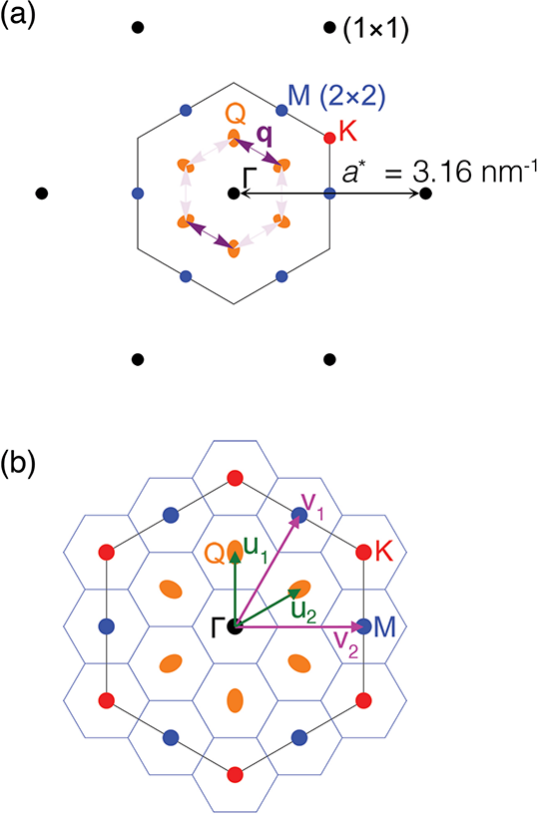
(a) Reciprocal lattice (black spots) and Brillouin zone with high symmetry points Γ, K, and M. Band minima at the low symmetry points, Q, lying half way along Γ–K, are shown. (b) Brillouin zone drawn with an overlaid (2√3 × 2√3) R30° unit cell. Vectors *u*_1_, *u*_2_ and *v*_1_, *v*_2_ indicate the Q and M points, respectively.

However, FSN cannot explain the appearance of the 2 × 2 periodicity in our STM data, because the electronic bands at the M-point are much higher in energy than the band minima.^7^ However, calculations of the MoS_2_ phonon band structure as a function of electron doping have demonstrated phonon softening at the M point, becoming imaginary for electron doping levels exceeding ∼0.14 electrons per molybdenum atom.^18,36^ The M point lies on the Brillouin zone boundary along the reciprocal lattice vector direction (Figure 4a) and as such is inherently associated with a 2 × 2 periodicity. This strongly suggests that momentum-dependent EPC can account for the formation of the 2 × 2 phase in K_0.4_MoS_2_.

Thus, our observations of a metal–insulator transition at 85 K, the opening of an ∼25 meV energy gap at the Fermi level, and the real space observation of a 2√3 modulation in STM and STS data provide compelling evidence for the existence of a FSN-driven CDW phase, where the nesting vector connects electron pockets at the Q points. This 2√3 phase is locally enhanced by defects in the MoS_2_ sheets that locally alter the interlayer coupling and enhances the electron pockets at the Q points. Simultaneously, we observe a nearly commensurate 2 × 2 modulation that matches a predicted 2 × 2 EPC-driven CDW due to phonon softening at the M point. As has been a feature of several previous reports of CDW phases in TMDs,^3,4^ it is not possible to fully describe all of our data with *either* a FSN *or* EPC model independently.

We suggest that the coexistence of the 2 × 2 and 2√3 phases is possible in part due to the effect of band folding. We illustrate the reciprocal space vectors of the 2√3 periodicity (*u*_1_ and *u*_2_) and 2 × 2 periodicity (*v*_1_ and *v*_2_) in Figure 4b, and it can be seen that these are simple linear combinations of one another (e.g., *v*_1_ = *u*_1_+*u*_2_). Thus, band folding established by the shorter periodicity (illustrated by the hexagonal tiling in Figure 4b) will result in the band minimum at Q and the soft phonon mode at M being mapped back onto the zone center, providing an opportunity for the two mechanisms to couple. The fact that the two modulations are simple linear combinations of one another also provides an explanation for why the Fermi level gap we measure in STS spectra contains 2 × 2 as well as 2√3 periodicity.

We have presented the discovery of a CDW ground state in K_0.4_MoS_2_, which is also the first observation of a CDW phase in a *d*^2^ (column 6) TMD. Owing to the unique band structure of K_0.4_MoS_2_ compared to *d*^0^ and *d*^1^ TMDs, our results provide new insight into the formation mechanisms of CDWs. We observe a metal–insulator transition at 85 K, an ∼25 meV energy gap centered at the Fermi level, and 2 × 2 and 2√3 periodicities that can be explained, respectively, by EPC and FSN mechanisms. The 2√3 periodicity is observed exclusively near defects, suggesting that the coexistence of FSN and EPC phases can be attributed to their delicate sensitivity to the band edge and the strength of the interlayer electronic coupling. Moreover, our observations suggest that CDW phases might be discoverable in other column 6 TMDs such as MoSe_2_, WS_2_, and WSe_2_^37^ at high electron-doping and for which investigation might provide further insight into the physics of CDW formation.

## Supporting Information Available

STM images of 2√3 modulations at defect sites acquired at 5.5, 10, and 77 K. STM images and analysis of phase slip boundaries. Spatially resolved tunneling spectroscopy of 2√3 modulation (PDF)

## Notes

The data created during this research are openly available via zenodo.org at 10.5281/zenodo.3696856.
