# Supplementary material for: Charge Density Waves in Electron-Doped Molybdenum Disulfide
Source: Nano Lett. 2021 Jul 6;21(13):5516–21. doi: 10.1021/acs.nanolett.1c00677 (PMC8397392; doi:10.1021/acs.nanolett.1c00677)
Supplement: Supplementary file 1 — nl1c00677_si_001.pdf [file nl1c00677_si_001.pdf]

SUPPLEMENTARY INFORMATION: Charge density waves in electron-doped molybdenum disulfide

Mohammed K. Bin Subhan,[1] Asif Suleman,[1,2] Gareth Moore,[1,2] Peter Phu,[2,3] Moritz Hoesch,[4,†] Hidekazu Kurebayashi,[2,3] Christopher A. Howard,[1] and Steven R. Schofield[1,2]

[1]Department of Physics and Astronomy, University College London, WC1E 6BT, London, UK

[2]London Centre for Nanotechnology, University College London, WC1H 0AH, London, UK

[3]Department of Electronic and Electrical Engineering, University College London, WC1E 6BT, London, UK

[4]Photon Science, Deutsches Elektronen-Synchrotron (DESY), Notkestrasse 85, 22607 Hamburg, Germany

[†]Previously at: Diamond Light Source, Didcot OX11 0DE, UK

June 19, 2021

Supplementary Data

Supplementary Figure S1 shows STM topographic images of three defects on $K_{0.4}MoS_2$ acquired at 77, 10, and 5.5 K, with their corresponding Fourier transform. The 1×1 surface sulfur lattice is clearly observed in each image, as well as the $(2\sqrt{3} \times 2\sqrt{3})R30^\circ$ modulation at the defect site. In each case, the spatial extent of the enhancement due to the defect is about 5 nm, and the $2\sqrt{3}$ modulation extends over the same distance, becoming weaker at distances further from the defect site. This behaviour contrasts to observations of defects in $NbSe_2$ [1], where the spatial extend of the 3×3 modulation varied from several nanometres, to covering the entire surface over a similar temperature range variation. We also note in Supplementary Figures. S1c and S1e that we observe no significant differences in the appearance of the surface above and below the expected superconducting transition temperature (~ 7 K).

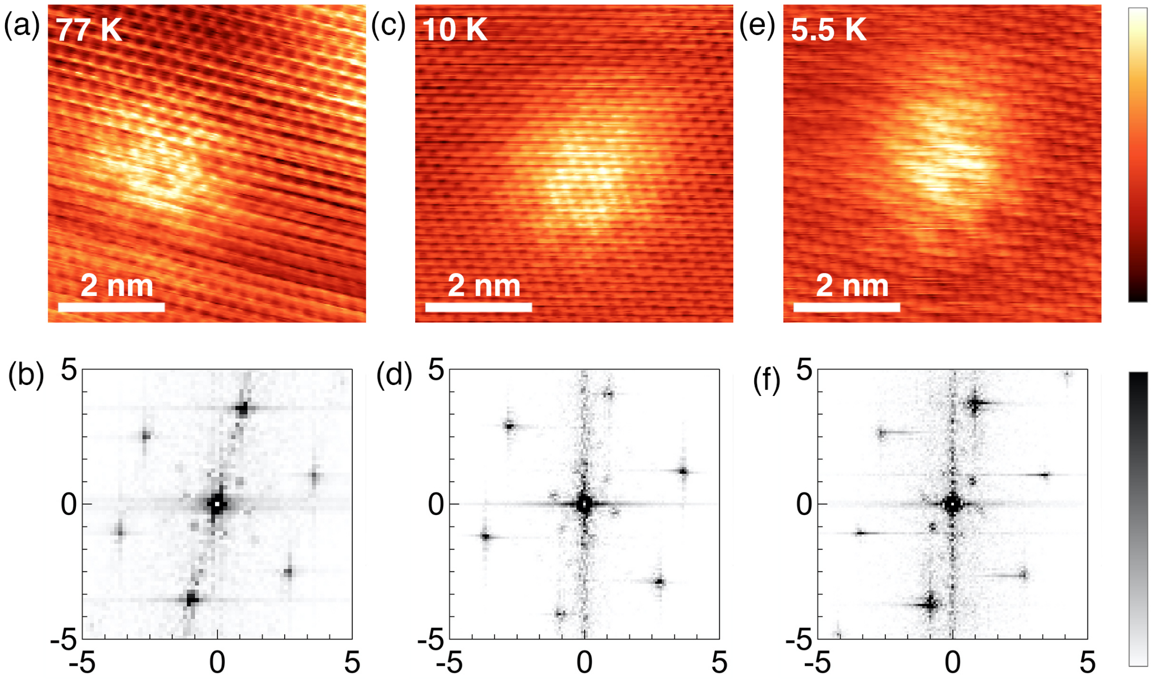

Supplementary Figure S1: STM topographic images of defects in $K_{0.4}MoS_2$ taken at (a) 77 K, (c) 10 K, (e) 5.5 K. The corresponding Fourier transforms are shown in panels (b), (d), and (f). Image parameters: (a) -400 mV, 50 pA, z-range 230 pm; (c) -100 mV, 30 pA, z-range 500 pm; (e) -100 mV, 30 pA, z-range 840 pm;

In the case that two defects occur in close proximity one another, we observe phase slip boundaries in the $2\sqrt{3}$ modulation, similar to the phase slip boundaries observed in the 3×3 CDW in $NbSe_2$ [2]. We illustrate this in Supplementary Figure S2(a), which shows a filled-state STM image of 10×10 nm^2 area where two defects are present. The 2D Fourier transform of this image is shown in Supplementary Figure. S2(b) and highlights that all three periodicities, 1×1, 2×2, and $2\sqrt{3}$, are all present in this image. Each of the two defects has an accompanying $(2\sqrt{3} \times 2\sqrt{3})R30^\circ$ modulation, but the two modulations are not in phase spatially, leading to a phase slip boundary between them, indicated by the white arrow. The phase change can be recognised by observing the hexagonal dark spot patterns in Supplementary Figure S2(a); as a guide to the eye we have drawn a blue rhombus to indicate the phase of the $2\sqrt{3}$ modulation at the bottom left defect site, and a green rhombus to indicate the $2\sqrt{3}$ phase of the top right defect.

In Supplementary Figures S2(c-e) we have use Fourier filtering to further highlight the features of this image. Supplementary Figure S2(d) has been low pass filtered to remove all of the regular periodicities in the image, leaving behind only the aperiodic background generated by the defect envelop function. The two bright protrusions in this image can be attributed to

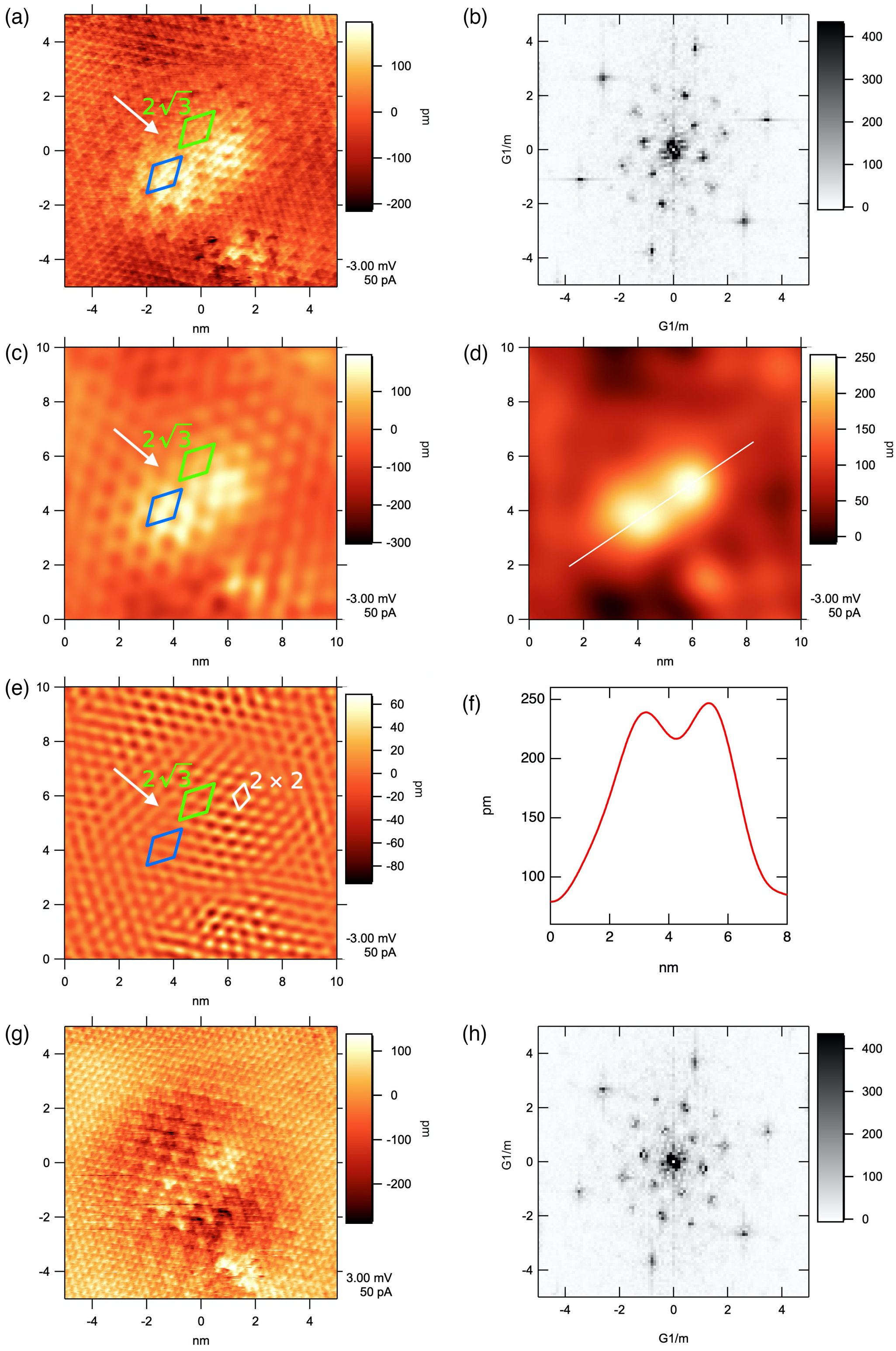

Supplementary Figure S2: (a) Filled-state STM image of a pair of closely-spaced defects in $K_{0.4}MoS_2$, with corresponding 2D Fourier transform shown in panel (b). 1×1, 2×2, and $2\sqrt{3}$ periodicities can be seen in the image and its Fourier transform. The arrow annotation on panel (a) highlights the location of a phase slip boundary in the $2\sqrt{3}$ modulation. (c-e) These three images are the same image from panel (a), that have been Fourier filtered as: (c) low-pass with a cutoff at 1.4 nm^{-1} to remove contributions other than the $2\sqrt{3}$ lattice and the very low frequency aperiodic contributions to the topography. (d) low-pass Fourier filtered with a cutoff at 0.8 nm^{-1}, which also removes the $2\sqrt{3}$ lattice and allows determination of the defect locations. (e) band filtered at six pockets around the 2×2 spots. (f) Line profile from image (d) showing the maxima of the defect envelope functions. (g) Empty-state STM image of the same area as panel (a). (h) Fourier transform of (g).

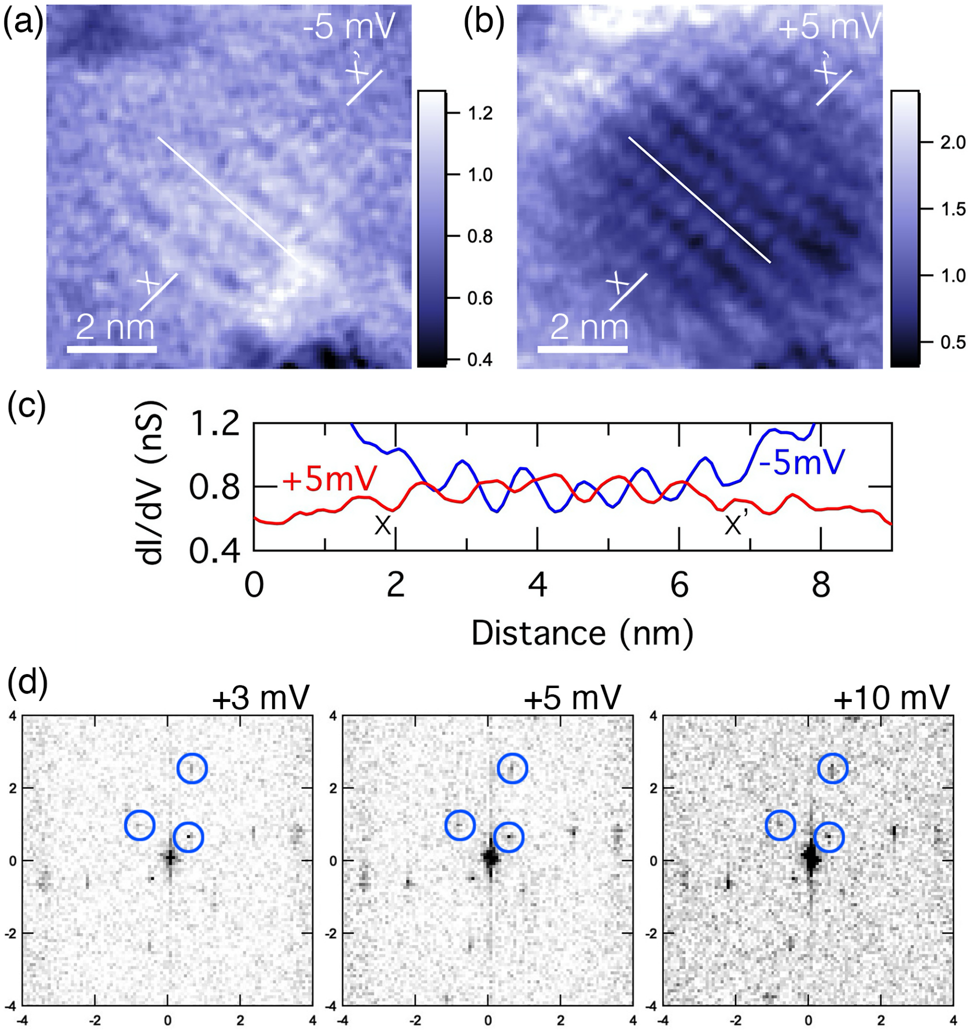

Supplementary Figure S3: (a,b) dI/dV maps (nS) at −5 meV and +5 meV, respectively and corresponding Fourier transforms. (c) Line profiles $x - x'$ as indicated in panels a and b. (d) Fourier transforms of the dI/dV maps at 3, 5, and 10 mV, highlighting that the superlattice periodicities are dispersionless.

the two individual defect sites. The line profile in Supplementary Figure S2(f) was taken across the defect to measure their separation, which was determined to be ~ 2.2 nm. Supplementary Figure S2(c) shows the filled state image from Supplementary Figure S2(a) low pass filtered slightly less aggressively such that the $2\sqrt{3}$ modulation still contributes to the filtered image and shows both the $2\sqrt{3}$ modulation and phase slip boundary clearly. We also notice in this image that, as with the other data presented, the intensity of the $2\sqrt{3}$ modulation decreases with distance from the centre of the defect. To illustrate the 2×2 lattice, we also Fourier filter Supplementary Figure S2(a) by the 2×2 spots, and the resulting image is shown in Supplementary Figure S2(e). This image shows that the 2×2 modulation extends over the entire image, with slightly varying intensity across the image, as expected for a nearly-commensurate CDW. It is also notable that no evidence of a phase slip boundary can be seen in the 2×2 modulation. Supplementary Figure S2(g) shows an empty-state image of the same region of the surface, which was acquired simultaneously with Supplementary Figure S2(a), and the corresponding 2D Fourier transform is shown in Supplementary Figure S2(h).

In Supplementary Figures S3(a,b) we show conductivity maps for $V = \pm 5$ mV, over an 8×8 nm^2 region of the surface where a single defect is present. Line profiles across this data (Supplementary Figures S3(c)) highlight both the protrusion/depression nature of the defect envelope [3], and a π phase shift in the $2\sqrt{3}$ modulation. This phase shift in the $2\sqrt{3}$ lattice between the occupied and unoccupied states is similar to previous observations in graphite [4], and is expected within a FSN CDW model. Supplementary Figure S3(d) shows Fourier transforms of the dI/dV maps at 3, 5, and 10 mV, highlighting the absence of dispersion.

References

1. Arguello, C. J. *et al.* Visualizing the charge density wave transition in 2H-NbSe2 in real space. *Phys. Rev. B* **89,** 235115 (2014).

2. Soumyanarayanan, A. *et al.* Quantum phase transition from triangular to stripe charge order in $NbSe_2$. *Proc. Natl. Acad. Sci.* **110,** 1623–1627 (2013).

3. Bampoulis, P. *et al.* Defect Dominated Charge Transport and Fermi Level Pinning in MoS2/Metal Contacts. *ACS Appl. Mater. Interfaces* **9,** 19278–19286 (2017).

4. Rahnejat, K. C. *et al.* Charge density waves in the graphene sheets of the superconductor CaC_6. *Nat. Commun.* **2,** 558 (2011).
